# *Chelatococcusthermostellatus* sp. nov., a new thermophile for bioplastic synthesis: comparative phylogenetic and physiological study

**DOI:** 10.1186/s13568-016-0209-9

**Published:** 2016-06-09

**Authors:** Mohammad H. A. Ibrahim, Liesbeth Lebbe, Anne Willems, Alexander Steinbüchel

**Affiliations:** Chemistry of Natural and Microbial Products Department, National Research Centre, Al-Bohoos st., Cairo, 12311 Egypt; Laboratory of Microbiology, Department of Biochemistry and Microbiology, Ghent University, K. L. Ledeganckstraat 35, 9000 Ghent, Belgium; Institut für Molekulare Mikrobiologie und Biotechnologie, Westfälische Wilhelms-Universität Münster, Corrensstrasse 3, 48149 Münster, Germany; Environmental Science Department, King Abdulaziz University, Jeddah, Saudi Arabia; Department of Chemistry and Chemical Biology, Center for Biotechnology and Interdisciplinary Studies, Rensselaer Polytechnic Institute, Troy, NY 12180 USA

## Abstract

The poly(3-hydroxybutyrate), PHB, accumulating thermophilic strain MW9^T^, isolated from an aerobic organic waste treatment plant, was characterized by detailed physiological and phylogenetic studies. The strain is a Gram-stain-negative, rod shaped, non-spore forming member of Alphaproteobacteria. It shows optimum growth at 50 °C. Based on 16S rRNA gene sequence similarity, the strain together with five very similar isolates, was affiliated to the genus *Chelatococcus* (Ibrahim et al. in J Appl Microbiol 109:1579–1590, 2010). Rep-PCR genomic fingerprints and partial *dnaK* gene sequence also revealed that these isolates are very similar, but differ from other *Chelatococcus* type strains. The major fatty acids were similar to those of other strains of the genus *Chelatococcus*. DNA–DNA hybridization of strain MW9^T^ with *Chelatococcus* species type strains revealed 11.0–47.7 % relatedness. G+C content of DNA was 67.1 mol%, which is comparable with the other strains of *Chelatococcus* species. The physiological and phenotypic characteristics of the new strain MW9^T^ are sufficient to differentiate it from previously described species in the genus *Chelatococcus*. Strain MW9^T^ is considered to represent a novel species of the genus *Chelatococcus*, for which the name *Chelatococcus thermostellatus* is proposed. The type strain is MW9^T^ (=LMG 27009^T^ = DSM 28244^T^). Compared to known *Chelatococcus* strains, strain MW9^T^ could be a potent candidate for bioplastic production at elevated temperature.

## Introduction

Increased global demands for alternative energy and biodegradable materials have stimulated many studies for the utilization of renewable resources via microbial fermentation (Chiellini and Corti 2012). Microbially synthesized biodegradable plastics, polyhydroxyalkanoates (PHAs), have been revealed to possess similar properties as conventional oil-based plastics, however industrial production costs of PHAs are relatively high (Chen 2010). Alternatively, biofunctionalized PHAs nanobeads can be used in numerous precious biotechnological applications (Dinjaski and Auxiliadora Prieto 2015). Moreover, its monomers are of great interest for biomedical applications, and could also be converted to alternative fuel (Chen 2010). Recently, fermentative production of PHA using thermophiles has gained more interest because of the possibility to reduce costs of sterilization and cooling during high-cell density cultivation at industrial scale (Ibrahim and Steinbüchel 2010).

Composting is a self-heating aerobic solid phase process for partial biodegradation and conversion of organic waste materials. A large variety of mesophilic, thermotolerant and thermophilic aerobic microorganisms have been reported to be predominant in composting at temperatures between 20 and 60 °C (De Bertoldi et al. 1983). Temperature is revealed to be a major factor determining the type of microorganism, species diversity, and the rate of metabolic activities during composting. It has been reported that at temperature of 50–60 °C, thermophilic bacteria are very active composting organisms (Beffa et al. 1995).

In a previous study of thermophilic bacteria, which are able to produce poly(3-hydroxybutyrate), PHB, the aerobic organic waste composting process in Münster (Germany) was used as a source to screen for new thermophiles. From different samples collected at various positions inside the compost heap, six thermophilic PHB-accumulating isolates, MW9^T^, MW10, MW11, MW12, MW13 and MW14 were purified and characterized (Ibrahim et al. 2010). Despite sharing over 99 % sequence similarity with each other in 16S rRNA genes (Ibrahim et al. 2010), these isolates showed some differences in aggregates formation, colony morphology, color, and size. It also showed some other biochemical and physiological characteristics differences such as carbon substrate preference. The 16S rRNA gene sequences of these isolates showed high similarities to those of *C. daeguensis* K106^T^ (98.96 %), *C. sambhunathii* HT4^T^ (98.84 %), and *C. asaccharovorans* TE2 (95.84 %) (Ibrahim et al. 2010).

The new isolates, except MW10, form star-shaped cell aggregates (SSC aggregates) during normal growth in mineral salts medium (MSM). They all grow optimally at 50 °C. These isolates show fast growth and shorter cell length without aggregation when grown in rich media; nutrient broth and standard I nutrient broth (Merck KGaA) (Ibrahim et al. 2010). Furthermore, in all these new *Chelatococcus* isolates, a growth-associated accumulation of intracellular PHB granules was observed when growing in medium with excess carbon source (Ibrahim et al. 2010).

In the present study, detailed physiological and molecular investigations were done for a precise differentiation of the new isolates from other *Chelatococcus* type strains. PHB accumulation and SSC aggregates formation by the new strain MW9^T^ and other strains of the genus *Chelatococcus* were also investigated.

## Materials and methods

### Isolation and cultivation of strains

Isolates MW9^T^, MW10, MW11, MW12, MW13 and MW14, were isolated from an aerobic compost of an organic waste treatment plant in Münster (Germany) (Ibrahim et al. 2010). Strains used for comparative study were *C. asaccharovorans* LMG 25503^T^, *C. daeguensis* LMG 25471^T^, *C. sambhunathii* LMG 26063^T^ (Table 3). The 16S rRNA gene sequences for isolates MW9^T^, MW10, MW11, MW12, MW13 and MW14 were deposited in the NCBI database under the accession numbers: GQ871852, GQ871853, GQ871854, GQ871855, GQ871856 and GQ871857, respectively.

Cultivations were done in three media: (i) Luria–Bertani broth (LB broth) (AppliChem GmbH, 64291 Darmstadt, Germany), (ii) standard-1-nutrient broth containing (g/L): peptone 15.0; yeast extract 3.0; sodium chloride 6.0; D(+)glucose 1.0 (Merck KGaA, 64271 Darmstadt, Germany); (iii) mineral salt medium (MSM, Schlegel et al. 1961) containing (g/L): Na_2_HPO_4_·12H_2_O, 9.0; KH_2_PO_4_, 1.5; MgSO_4_·7H_2_O, 0.2; NH_4_Cl, 1.0; CaCl_2_·2H_2_O, 0.02; Fe(III)NH_4_-citrate, 0.0012. The MSM also included 1 mL of trace element solution containing the following compounds (g/L): EDTA, 50.0; FeCl_3_, 8.3; ZnCl_2_, 0.84; CuCl_2_·2H_2_O, 0.13; CoCl_2_·6H_2_O, 0.1; MnCl_2_·6H_2_O, 0.016; H_3_BO_3_, 0.1. Glucose (20 g/L) was added separately as sole carbon source. The pH of the medium was adjusted to 7.3 before sterilization. Agar (16 g/L) and/or Nile-red (final concentration 0.5 µg/mL), was added for solid media preparation.

### Cultures for PHA accumulation

Erlenmeyer flasks (250 mL) containing 50 mL MSM with glucose (20 g/L) were inoculated with 3.5 mL of a 24-h grown pre-culture (7 % inoculum size). The pre-culture was inoculated with a full-loop of well-grown culture on MSM. Unless stated otherwise, flasks were incubated at 50 °C and 200 rpm for 72 h. Cells were harvested by centrifugation for 20 min at 3072×*g* and 4 °C, washed with distilled water, frozen and lyophilized.

### Gas chromatography analysis of PHA content

PHA content in whole cell dry matter was determined by gas chromatography analysis after methanolysis and 3-hydroxybutyric acid methyl ester formation (Ibrahim et al. 2010).

### Scanning electron microscopy (SEM)

Samples were fixed in 0.15 M cacodylate buffer (2.5 % glutaraldehyde) overnight. The samples were attached to poly-l-lysine prepared cover slips, alcohol dehydrated and critical point dried. Then they were mounted on aluminum and sputter coated with gold/palladium.

### Transmission electron microscopy (TEM)

The cells were fixed in 0.1 M sodium cacodylate buffer (3 % glutaraldehyde) overnight, postfixed in 1 % osmium tetroxide for 1 h and dehydrated in ethanol. They were embedded in Epon (Taab), 50 nm thick sections were cut off with a diamond knife and stained with 2 % uranyl acetate and lead citrate, according to Reynolds (1963), and examined in a JEOL JEM 1230 transmission electron microscope.

### Rep-PCR analysis

For rep-PCR, DNA preparations were made starting from one or two colonies of each isolate/strain and using the alkaline lysis protocol of Baele et al. (2003). Rep-PCR was performed as described by Gevers et al. (2001) using the (GTG)_5_ primer (5′-GTG GTG GTG GTG GTG-3′), and the resulting genomic fingerprints were processed using BioNumerics v. 5.1 software (Applied Maths).

### Sequence analysis of *dnaK*

The same DNA preparations as used for rep-PCR were also used to amplify *dnaK* genes using the primers and protocol described previously (Stępkowski et al. 2003; Martens et al. 2007). Sequence analysis was performed using MEGA version 5 (Tamura et al. 2011). Using Muscle in MEGA, *dnaK* gene sequences of the new isolates were aligned with those of the related strains. A neighbor-joining tree was calculated and a bootstrap analysis using 1000 replications was performed.

### Fatty acid analysis

Whole-cell fatty acid composition was analyzed for strain MW9^T^, as well as for the type strains of the three other *Chelatococcus* species. All strains were grown for 48 h on TSA (LMG medium 185) at 37 °C. Harvesting of cells, extraction and analysis were performed according to the recommendations of the manufacturer of the MIDI identification system (Microbial Identification System). The fatty acid methyl esters mixtures were separated using MIDI SHERLOCK Microbial Identification System (Microbial ID, Newark, DE, 19711, USA.) and an Agilent model 6890 series gas chromatograph fitted with a 7683 automatic sample injector. Peaks were assigned using the Sherlock MIS software and the TSBA50 peak naming method.

### DNA–DNA hybridization

High-quality DNA for DNA–DNA hybridization was prepared by the method of Wilson (1987), with minor modifications (Cleenwerck et al. 2002). DNA–DNA hybridization was performed using the microplate method (Ezaki et al. 1989) with some modifications as described by Cleenwerck et al. (2002). The hybridization temperature was 45 ± 1 °C. Reciprocal reactions were performed for every hybridization pair and variation was within the limits of this method (Goris et al. 1998). The values presented are based on a minimum of four replicates (Table 3).

### G+C content

The G+C content of DNA was determined by HPLC according to the method of Mesbah et al. (1989) using a Waters Breeze HPLC system and XBridge Shield RP18 column thermostabilised at 37 °C. The solvent was 0.02 M NH_4_H_2_PO_4_ (pH 4.0) with 1.5 % (v/v) acetonitrile. Non-methylated lambda phage (Sigma) and *E. coli* DNA were used as calibration reference and control, respectively.

## Results

### Morphological and physiological characterization

Cell shape of all *Chelatococcus* type strains varies from rods to coccoid rods (Table 1). The isolates grew optimally at 50 °C and accumulate high content of PHB (Ibrahim et al. 2010), whereas the other type strains of genus *Chelatococcus* did not grow at the temperature being investigated in this study (50 °C).

**Table 1:** Differential characteristics between strains MW9^T^ and the type strains of recognized *Chelatococcus* species

	*C. thermostellatus* MW9^T^ DSM 28244^T(a)^	*C. asaccharovorans* DSM 6462^T (a)^	*C.* *daeguensis* K106^T^ CCUG 54519^T (a)^	*C. sambhunathii* HT4^T^ DSM 18167^T (a)^
Isolation source	Organic waste compost (Ibrahim et al. 2010)	Wastewater, soil (Auling et al. 1993)	Textile waste matter (Yoon et al. 2008)	Hot spring (Panday and Das 2010)
Cell morphology	Rods	Coccoid rods	Rods^(b)^	Short rods^(b)^
Motility	+	−	+^(b)^	+
Mucoid colony on MSM with glucose	−	ND	+^(b)^	−^(b)^
Hydrolysis of gelatin	+	+	−	+
Use of NTA as sole carbon and nitrogen source	−	+	−	+
Utilisation of				
Mannose, gluconate	+	+	+	−
Xylose	−	+	+	−
Mannose	+	+	+	−
Glycerol	w	−	−	−
Sucrose	w	−	−	−
Growth temp.				
Range	37–55 °C	4–41 °C	20–51 °C	20–50 °C
Optimum	50 °C^(b)^	35–37 °C	30–37 °C^(b)^	37–42 °C^(b)^
Growth pH				
Range	6.0–8.5	5.5–9.5	5.5–10.0	6.0–8.5
Optimum	7.0–7.5	7.0–8.0	7.0–7.5	7.5–8.0
Star-shaped aggregates	+^(b)^	−	+^(b)^	+^(b)^
PHB granules	+^(b)^	+	+^(b)^	+^(b)^

Data on assimilation of carbon sources are from Ibrahim et al. (2010) and from Panday and Das (2010)

*+* positive, *−* negative, *w* weak, *ND* not determined

^a^Data are from (Ibrahim et al. 2010), (Panday and Das 2010) or related references

^b^Data confirmed by experiments done in the present study

**Fig. 1:**
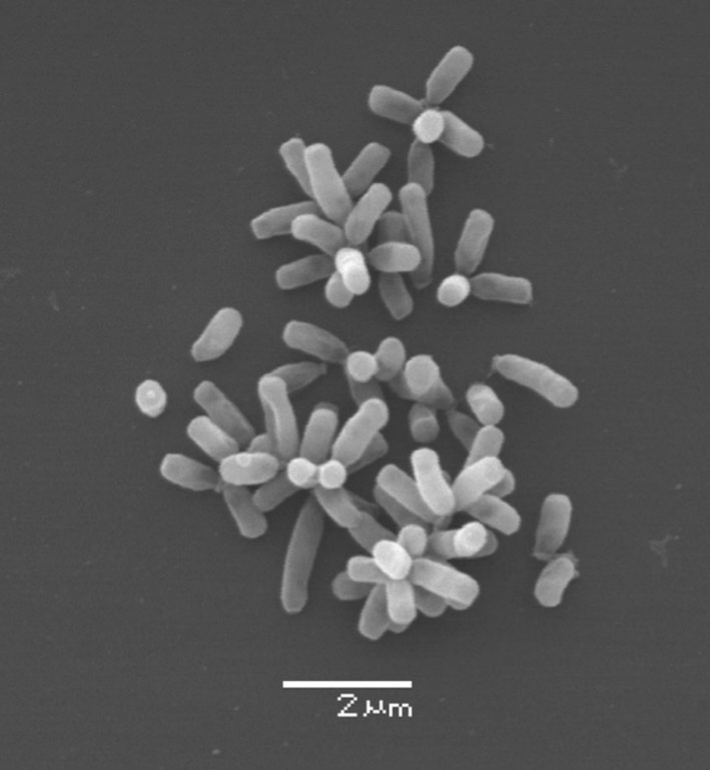
Scanning electron microscopy (SEM) show the star-shaped cell aggregates (SSCAs) formed by the strain MW9^T^

One of the main characteristics for the new isolates was the formation of stable star-shaped cell aggregates during growth in MSM with glucose or glycerol (Ibrahim et al. 2010), (Fig. 1). These aggregates were not reported before for strains of the genus *Chelatococcus*. In a comparative experiment, type strain *C. daeguensis* LMG 25471^T^ and *C. sambhunathii* LMG 26063^T^ did show star or rosette-shaped cell aggregates, respectively, during growing in MSM with glucose at temperature 37 °C.

### PHA accumulation

**Fig. 2:**
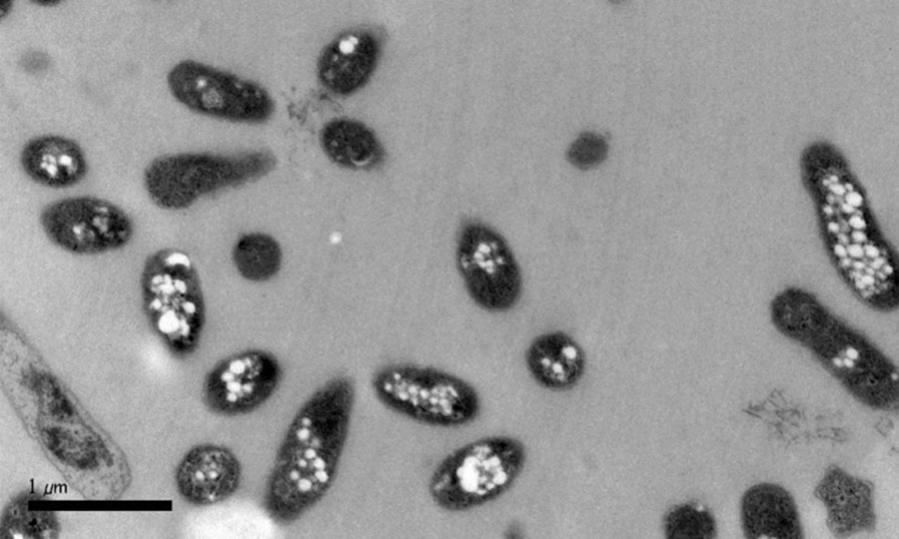
Transmission electron microscopy (TEM) for cells of strain MW9^T^. Intracellular granules of PHB are distinguished inside cells of MW9^T^

A detailed investigation of PHA synthesis by the strains *C. daeguensis* LMG 25471^T^ and *C. sambhunathii* LMG 26063^T^ was conducted in the present study. Cultures were inoculated in MSM supplemented with 20 g/L glucose (250-mL Erlenmeyer flask with 50 mL medium) and incubated at 37 °C and 200 rpm for 60 h. Gas chromatography analysis of the PHA content in the lyophilized cells revealed that strains *C. daeguensis* LMG 25471^T^ and *C. sambhunathii* LMG 26063^T^ were able to accumulate the homopolymer poly(3-hydroxybutyrate) from glucose to a content of 44.5 ± 1.5 % of cell dry weight, CDW, [3.29 g CDW/L (0.165 g biomass/g glucose)] and 54.4 ± 0.3 % of CDW [4.83 g/L (0.242 g biomass/g glucose)], respectively. In comparison, a higher PHB content was detected in strain MW9^T^ [73.0 ± 2.3 % of CDW (3.94 g CDW/L, yield equivalent to 0.197 g biomass/g glucose), Fig. 2] in the same medium, however at higher incubation temperature (50 °C). Bioplastic (PHB) productivity and yield by strain MW9^T^ is the highest between studied *Chelatococcus* strains [2.88 g/L PHB (0.144 g PHB/g glucose), versus 2.63 g/L PHB (0.131 g PHB/g glucose), 1.46 g/L PHB (0.073 g PHB/g glucose by the other strains), respectively].

### Rep-PCR genomic fingerprints analysis

**Fig. 3:**
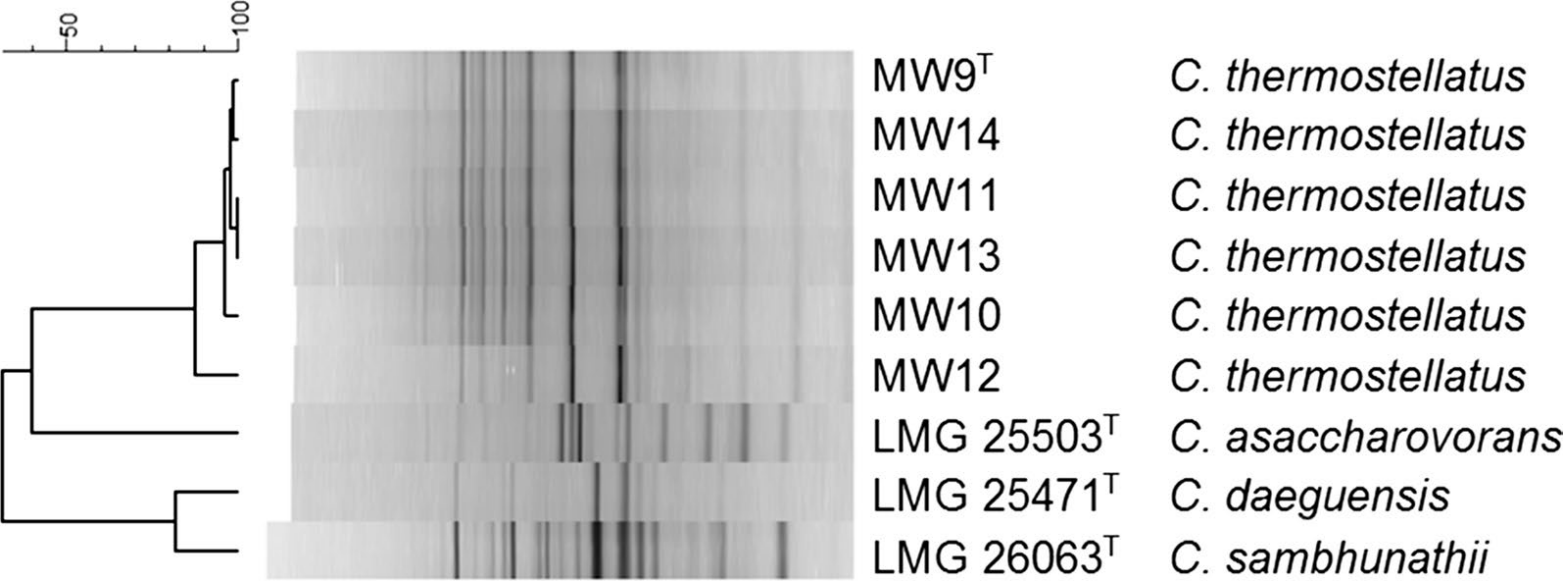
Comparison of genomic fingerprints obtained by rep-PCR. Profiles were compared using Pearson product moment correlation coefficients and UPGMA clustering using BioNumerics

Rep-PCR genomic fingerprints shown in Fig. 3. demonstrate that the different isolates obtained from an aerobic organic solid waste treatment plant using enrichment technique in MSM with different carbon sources (Ibrahim et al. 2010), are very similar, but differ from other *Chelatococcus* species.

### Partial *dnaK* gene sequences comparison

**Fig. 4:**
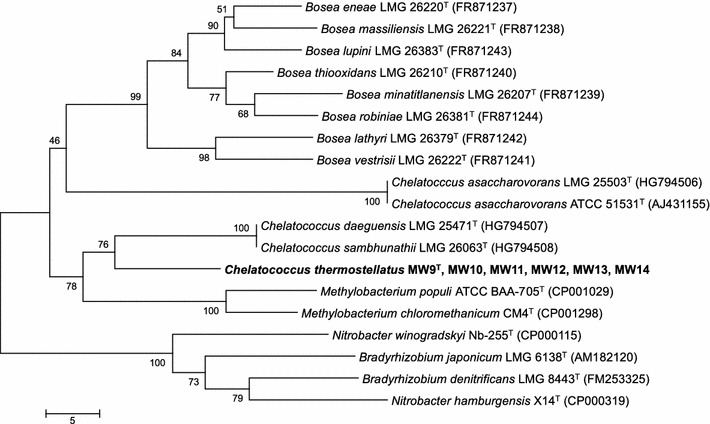
Phylogenetic tree based on neighbor-joining analysis of partial *dnaK* gene sequences showing the relatedness of *Chelatococcus* isolates and closest neighbors. Partial *dnaK* gene sequence of strain MW9^T^ was identical to MW10, MW11, MW12, MW13 and MW14 gene sequences. Bootstrap values expressed as percentages of 1000 replicates are shown at the branch nodes. *Scale bar*, five estimated base differences per sequence

Partial *dnaK* gene sequences (280 bp) of all six new isolates were identical (Fig. 4), (all sequences of the new isolates clustered together and are represented by strain MW9^T^), and showed sequence similarities of 87.5 % with those of the type strains of *C. daeguensis* and *C. sambhunathii* and 76.8 % with the type strain of *C. asaccharovorans*. The new isolates formed a phylogenetic cluster with the type strains of *C. daeguensis* and *C. sambhunathii* with 76 % bootstrap support (Fig. 4), while the type strain of *C. asaccharovorans* was grouped separately.

Phylogenetic trees, updated from our 2010 study (Ibrahim et al. 2010), are available as supplementary figures (S1, 16S rRNA gene sequences Maximum Likelihood tree; S2, 16S rRNA gene sequences Neighbor Joining tree; S3, 16S rRNA gene sequences Maximum Parsimony tree; S4, partial *dnaK* gene sequences Maximum Likelihood tree). Because of the identity between the new isolates, only the isolate MW9^T^ was selected as type strain and as a representative for further molecular characterization.

### Utilization of nitrilotriacetic acid (NTA)

Isolate MW9^T^ was investigated for NTA utilization as previously reported for the type strain *C. asaccharovorans*. Cells were cultivated in MSM (1 g/L NH_4_Cl) supplemented with glucose (20 g/L) at the standard conditions in comparison with growth in the same medium with NTA, but without both nitrogen and carbon sources. Isolate MW9^T^ could not grow in the NTA-containing medium even after 4 days of incubation.

### Fatty acid analysis

The new strain MW9^T^ did not grow at the standard temperature of 28 °C. Its growth temperature range was previously determined to be 37–55 °C with an optimum of 50 °C, however, at this temperature the *Chelatococcus* reference strains failed to grow. The reference strains did grow at 37 °C, and therefore this temperature was used for all strains to allow comparison.

The fatty acid profiles of all species (Table 2) were rather similar. Major fatty acids (>10 %) were 18:1 ω7c, 19:0 cyclo ω8c and Summed Feature 2, which may comprise 12:0 aldehyde, 16:1 iso I and/or 14:0 3OH that could not be separated by the MIDI system. Other fatty acids present in all species but in lower quantities were 16:0, 17:0, 18:0 and 18:0 3OH. Some fatty acids did show a quantitative difference in the different strains (Table 2) although with just one strain representing each species, these differences may be not very diagnostic.

**Table 2:** Fatty acid composition of strain MW9^T^ and the type strains of *Chelatococcus* species, grown for 48 h on TSA (LMG medium 185) at 37 °C

Fatty acid	*C. thermostellatus*	*C. asaccharovorans*	*C. daeguensis*	*C. sambhunathii*
	MW9^T^	LMG 25503^T^	LMG 25471^T^	LMG 26063^T^
11 Methyl 18:1 ω7c	–	0.2	–	–
14:00	–	0.3	–	–
15:0 3OH	0.4	0.1	–	–
16:00	2	10.9	4.5	4.6
16:0 3OH	–	0.8	–	–
17:00	0.9	0.8	3.6	4.4
17:0 Cyclo	0.2	1.7	–	–
18:00	4.1	3.1	4.4	3.2
18:0 3OH	5.2	2.8	4.8	2.2
18:1 2OH	8.3	–	3.4	6.8
18:1 ω7c	38.8	18.4	40	35.3
19:0 Cyclo ω8c	27.8	48.7	31.4	25.4
19:0 10 Methyl	0.3	–	–	–
20:1 ω7c	1	1.4	–	–
20:2 ω6,9c	–	0.1	–	–
Summed feature 1	0.5	–	–	1.5
Summed feature 2	10.1	10.1	7.2	16.5
Summed feature 3	0.2	–	–	–
Unknown ECL 14.502	–	0.2	–	–
Unknown ECL 14.959	0.4	–	0.7	–

Summed feature 1 comprises 15:1 ISO I/H and 13:0 3OH; Summed feature 2 comprises 12:0 aldehyde, 16:1 iso I and/or 14:0 3OH; Summed feature 3 comprises 16:1 w7c and 15 iso 2OH

– not detected

### G+C content and DNA–DNA homology study

The G+C content of strain MW9^T^ DNA (67.1 mol%) and *Chelatococcus* type strains are similar and ranged from 64.0 to 67.8 mol% (Table 3).

**Table 3:** DNA G+C content and DNA–DNA relatedness between strain MW9^T^ and *Chelatococcus* species

Strain	DNA G+C content (mol%)	DNA–DNA relatedness (%)		
		MW9^T^ LMG 27009^T^	LMG 25503^T^	LMG 25471^T^
*C. thermostellatus* MW9^T^ LMG 27009^T^	67.1			
*C. asaccharovorans* LMG 25503^T^	67.7	11.0 ± 2.8		
*C. daeguensis* LMG 25471^T^	64.0	47.7 ± 5.8	12.1 ± 1.8	
*C. sambhunathii* LMG 26063^T^	67.8	42.5 ± 4.2	4.9 ± 0.6	22.1 ± 1.7

Low level of DNA–DNA hybridization was detected between strain MW9^T^ and *C. asaccharovorans* LMG 25503^T^ (11.0 %), *C. daeguensis* LMG 25471^T^ (47.7 %), and *C. sambhunathii* LMG 26063^T^ (42.5 %).

## Discussion

The genus *Chelatococcus* was first described by Auling et al. (1993) and later by Egli and Auling (2005) as consisting of gram negative bacteria with the distinctive characteristics of utilizing the metal-chelating aminopolycarboxylic acid nitrilotriacetic acid (NTA). Following the first description of species *C. asaccharovorans* (Auling et al. 1993; Egli and Auling 2005), two distinct species were recently affiliated to the genus, *C. daeguensis* (Yoon et al. 2008) and *C. sambhunathii* (Panday and Das 2010). Ecologically, strains of the genus were isolated from different aquatic and waste treatment environments (Wilberg et al. 1993; Egli and Auling 2005; Ibrahim et al. 2010; Xu et al. 2014).

New physiological characteristics were reported for the new isolates, MW9^T^, MW10, MW11, MW12, MW13 and MW14, such as high optimum growth temperature, star-shaped cell aggregates, and PHA accumulation. The 16S rRNA gene sequences of these isolates are very similar and place them in the genus *Chelatococcus* (Ibrahim et al. 2010).

These new unusual characteristics made it important to conduct a more detailed study for a precise affiliation of these isolates within the genus *Chelatococcus*, possibly as a new species. Moreover, a clear differentiation between the new isolates is needed.

SSC aggregates have previously been found in several taxa within the Alphaproteobacteria (Biebl et al. 2007). However, this aggregation was not previously reported for any of the *Chelatococcus* species, possibly because growth was usually studied in nutrient-rich media. In the present study and during cultivation in mineral salts medium, the type strains *C. daeguensis* LMG 25471^T^ and *C. sambhunathii* LMG 26063^T^ showed SSC aggregation during growth.

Little information about PHA accumulation was reported for strains belonging to related *Chelatococcus* species: PHB granules were observed in the type strain of *C.asaccharovorans* (Egli and Auling 2005) (Table 1) and very recently, a new thermophilic denitrifying bacterial strain of *C. daeguensis*, TAD1, was reported to accumulate poly(3-hydroxybutyrate) at 45 and 50 °C from glucose (Xu et al. 2014). PHA accumulation can be regarded as a characteristic attribute of the genus *Chelatococcus*, especially after the confirmation of high PHB content in cells of strains *C. daeguensis* LMG 25471^T^ and *C. sambhunathii* LMG 26063^T^ in the present study, and the high content reported for our new thermophilic isolates (Ibrahim et al. 2010).

The identity of rep-PCR profiles and partial *dnaK* gene sequences between the new isolates together with the high similarity of their 16S rRNA gene sequences would suggest that they may not be classified as separate organisms, but rather be different colonies with slightly differences in carbohydrates preference (Ibrahim et al. 2010). We therefore chose the glucose utilizing isolate MW9^T^ as representative of the new thermophilic isolates and describe it as a new species given the differences in 16S rRNA, rep-PCR, and partial *dnaK* gene sequences with other *Chelatococcus* species. This isolate was used for further investigations.

The *dnaK* gene sequence of the type strain of the type species *C. asaccharovorans* showed less similarity to all new *Chelatococcus* strains (Fig. 4), even though it did belong to the *Chelatococcus* cluster in 16S rRNA gene phylogeny (Ibrahim et al. 2010), indicating its *dnaK* sequence may be affected by lateral gene transfer. Comparison of the complete *dnaK* gene might clarify this possibility.

Utilization of NTA as the sole carbon and nitrogen source was investigated for the new strain MW9^T^. However, this strain was not able to grow on NTA alone. Similar results were also reported for strain *C. daeguensis* (Yoon et al. 2008). The emended genus description by Yoon et al. (2008) also stated that this characteristic could be positive or negative variable within different species of *Chelatococcus*.

DNA G+C content, together with the physiological properties summarized in Table 1, are in accordance with the genus amendment of *Chelatococcus* reported by Yoon et al. (2008). Considering the 70.0 mol% DNA–DNA relatedness cut-off point recommended for bacterial species delineation (Wayne et al. 1987), strain MW9^T^ should be regarded as representing a novel species of the genus *Chelatococcus*.

The phylogenetic distinctiveness and low DNA–DNA relatedness with other *Chelatococcus* type strains reported here as well as phenotypic differences (Table 1), justify the creation of a new *Chelatococcus* species for strain MW9^T^, for which the name *Chelatococcusthermostellatus* sp. nov. is proposed. The species was isolated from aerobic organic waste compost in Germany, and DNA sequence data indicate it may be present in other compost systems. Indeed, a FASTA search with the 16S rRNA gene sequence of MW9^T^ in the EMBL database Environmental subsection revealed highly similar *Chelatococcus* sequences from asparagus straw compost from China (99.9 %, accession number JQ740271, clone ASC373), from a pilot scale municipal drum compost from Finland (99.8 %, FN667527, clone PS3657), from dairy manure and rice chaff composting samples in China [99.6 %, JQ337129, clone NT-3-59 (Tian et al. 2013)], and from fermented composting material in China [99.3 %, FJ930065, clone 6 (Wang et al. 2011)].

As a conclusion, the present study, while presenting a new species in the genus *Chelatococcus*, also confirmed PHA accumulation as a new genus-wide feature and showed that the formation of SSC aggregates can be used to describe many strains of *Chelatococcus* species. Strain MW9^T^ is capable of growing and producing high content of PHB at higher temperature compared to other related strains. This makes this new *Chelatococcus* strain a potent candidate for bioplastic production at thermophilic condition, where production costs can be minimized. Further physiological and proteomic investigations should facilitate a precise differentiation between isolates, MW9^T^, MW10, MW11, MW12, MW13 and MW14.

### Description of *Chelatococcus thermostellatus* sp. nov

*Chelatococcus thermostellatus* [ther.mo.ste’llatus. Gr. adj. *thermos*, hot; N.L. v. *stellatus,* to form star-shape cell aggregates; N.L. part. adj. *thermostellatus*, referring to the formation of star-shaped cell aggregates at high temperatures].

Cells are Gram-negative, oxidase positive motile rods (0.2–0.4 × 1.0–2.5 μm), aerobic, and non-spore forming. Good growth occurs on LB broth, nutrients broth, standard I nutrient broth, and MSM-gluconate/glucose/glycerol after 1–3 days at 50 °C. No growth was detected below 37 °C or above 55 °C. Optimum pH 7.0–7.5. NTA was not utilized as a sole source for carbon and nitrogen. During optimum growth on MSM, star-shaped cell aggregates, and PHB intracellular granules were observed as well. Colonies on MSM are circular with bright white to beige color and 0.5–1.0 mm in diameter. The major fatty acids comprises (>10 %) 18:1 ω7c, 19:0 cyclo ω8c and summed feature 2 (12:0 aldehyde, 16:1 iso I and/or 14:0 3OH). The DNA G+C content of the type strain is 67.1 mol%.

The type strain MW9^T^ (=LMG 27009^T^ = DSM 28244^T^) was isolated from aerobic organic waste compost in Münster, Germany.
